# Finite Element Simulation and Microstructural Analysis of Roll Forming for DP590 High-Strength Dual-Phase Steel Wheel Rims

**DOI:** 10.3390/ma17153795

**Published:** 2024-08-01

**Authors:** Jingwen Song, Jun Lan, Lisong Zhu, Zhengyi Jiang, Zhiqiang Zhang, Jian Han, Cheng Ma

**Affiliations:** 1 School of Materials Science and Engineering, Tianjin University of Technology, Tianjin 300384, China; sjw13314701971@163.com (J.S.); lj526586@163.com (J.L.); 2 School of Mechanical, Materials, Mechatronic and Biomedical Engineering, University of Wollongong, Wollongong, NSW 2522, Australia; lz131@uowmail.edu.au (L.Z.); jiang@uow.edu.au (Z.J.); 3 Han-Steel, Handan 056015, China; zzq198265@163.com; 4 Technology Research Institute, HBIS Group, Shijiazhuang 050023, China

**Keywords:** flash butt welding, dual-phase steel DP590, FE modeling, roll forming

## Abstract

In this study, finite element (FE) simulation by the software Abaqus was relied on to investigate the roll forming process of a wheel rim made of an innovative dual-phase steel, i.e., DP590, after flash butt welding (FBW). In the simulation, an FE model was generated, including the design of the dies for flaring, three-roll forming, and expansion, and detailed key processing parameters based on practical production of the selected DP590. Combined with the microstructures and properties of the weld zone (WZ) and heat-affected zones (HAZs) after FBW, the distribution of stress/strain and the change in thickness of the base metal (BM), WZ and HAZs were analyzed, and compared in the important stages of roll forming. Theoretically, the variation in the microstructure and the corresponding stress–strain behaviors of the BM, WZ, and HAZs after FBW have led to the thickness reduction of DP590 that originated from softening behaviors occurring at the region of subcritical HAZs (SCHAZs), and a small amount of tempered martensite has evidently reduced the hardness and strength of the SCHAZ. Meanwhile, the distribution of stress/strain has been influenced to some extent. Further, the study includes the influence of the friction coefficient on the forming quality of the wheel rim to guarantee the simulation accuracy in practical applications. In sum, the dual-phase steel has to be carefully applied to the wheel rim, which needs to experience the processes of FBW and roll forming, focusing on the performance of SCHAZs.

## 1. Introduction

**Figure 1:**
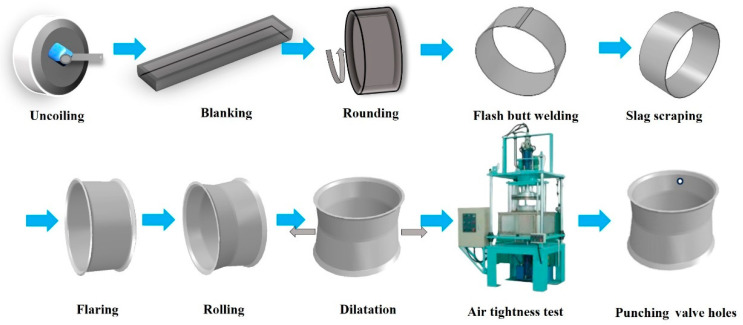
Rim production process diagram.

As an important component, the wheel rim plays an important role in the smooth and safe operation of automobiles [1]. The rim and the tire support each other and buffer the force transmitted by the ground, so the performance of the entire wheel is largely determined by the performance of the rim. With the rapid development of the manufacturing industry, various research and development institutes have carried out a series of technical studies and research on steel wheels to improve the strength, fatigue, and other mechanical properties of the wheel rim, as well as the comprehensive performance of welding and forming, to ensure the safety of the wheel rim [2,3]. Modern tubeless rims generally use equal-thickness steel plates as raw materials [4], with a 2.5–8 mm thickness range. The general process of their production isinvolves (1) uncoiling, (2) blanking, (3) rolling, (4) flash butt welding, (5) slag scraping, (6) flaring, (7) one-pass rolling, (8) two-pass rolling, (9) three-pass rolling, (10) expansion and finishing, (11) air tightness test, and (12) punching valve holes. The process diagram is shown in Figure 1.

The rim material is made using high-strength steel DP590. Dual-phase (DP) steel is a low-carbon steel alloy subjected to critical-zone heat treatment or a controlled rolling process to obtain martensite and ferrite at a faster cooling rate [5]. Since the dual-phase steel has a hardened phase distributed on the fine ferrite matrix and continues to be strengthened by solid solution atoms [6], 5–20% martensite guarantees the strength of the material. With the increase in martensite content, the strength of DP steel can reach 500–1200 MPa [7,8].

There is no obvious yield point in the stress–strain curve of DP steel, which effectively avoids the wrinkling problem on the surface of the formed parts. DP steel has a low yield strength and high tensile strength, which makes it have a high yield ratio. The strength of the workpiece during the forming process is low, and the strength after forming is high. Therefore, the formed parts have high impact energy absorption and fatigue strength. During the deformation process, the elongation of DP steel is uniform and the total elongation is large [9]. Therefore, dual-phase steel has good comprehensive mechanical properties and is a potential material for rims [10].

Flash butt welding technology is the mainstream method for rail welding. In addition to being used in the field of rails, it is also used for the closed welding of open blanks and parts, such as automobile rims, bicycle rims, and motorcycle rims [11]. Because it has no need to add other materials [12] and has a high welding efficiency, it has become the mainstream welding method for wheel rim production. In the flash butt welding process, high-temperature plastic deformation occurs in the weld and the heat-affected zone near the weld, which leads to a change in the microstructure and mechanical properties [13], thus affecting the subsequent forming process. Therefore, the roll-forming quality of welded joints is closely related to the welding process parameters [14,15].

Compared with the traditional design method, numerical simulation technology has great advantages in terms of cost, efficiency, and working environment. More and more mechanical manufacturing processes have been unprecedentedly developed with the support of finite element technology [16]. A reasonable finite element model is established to provide scientific researchers and engineers with the dynamic details of the product during processing [11]. The important processes in rim production were studied, and the influence law of key process parameters was found and was reasonably regulated, which can improve product quality, shorten the development cycle, and cut costs [17].

There has been a lot of research on the numerical simulation of the roll forming process, and some experience has been summarized. Kiuchi [18] developed a computer-based numerical simulation system for cold roll forming, which is suitable for various roll forming processes. It is convenient to analyze the influence of rolling pass, roll position, product size, and sheet mechanical properties on forming. Abe et al. [19] used the rigid-plastic finite element method to simulate the multi-pass forming of spokes, discussed the thinning of wall fillet thickness in each stage, and studied the influence of fillet punch radius and tensile ratio on fillet thinning. Bi et al. [20] carried out a numerical simulation of the spinning of automobile rims and improved the spin forming process. The research results have a certain reference value for the study of rim spin forming. Fang et al. [21] established a finite element model of the rolling process in ABAQUS, studied the law of rim roll forming, and analyzed the deformation results of each pass. The reliability of the model was verified, which provides a reference for the numerical simulation of high-precision rims. Finally, the problem of rim cracking was analyzed, and the influence of different flaring die angles on the forming results of the rim was explored. Lu et al. [22] established a simulation model of rim plastic forming, studied some key technologies of the model, analyzed the distribution of stress and strain, and compared it with the actual production of the rim to verify the accuracy of the model. Hwang et al. [23] used an offline hot-rolling simulator and numerical simulation technology to simulate and analyze the measured temperature of each region of the workpiece.

In comparison to existing research, this study focuses on DP590 steel wheel rims and utilizes ABAQUS 2020 finite element analysis software to establish models to investigate the key mechanisms involved in the forming process of wheel rims post-flash welding. The study integrates the processes of flash welding and roll forming, delineating the welding seam and heat-affected zone within the model. Through microscopic structural analysis, it correlates the stress–strain distribution and thickness during roll forming with the actual microstructure, revealing the association between microstructural changes and macroscopic forming quality. The study explores the impact of critical process parameters on this process to ensure the accuracy of simulation results in practical applications. The findings of this research are crucial for optimizing wheel rim manufacturing processes, enhancing product quality, and reducing production costs. They provide new insights into improving wheel rim roll forming processes and serve as a reference for future studies.

## 2. FEM Model of the Roll Forming of a Wheel Rim

### 2.1. Die Design

The research object of this study is DP590, and its alloy composition and mechanical properties parameters, obtained by tensile test, are shown in Table 1.

**Figure 2:**
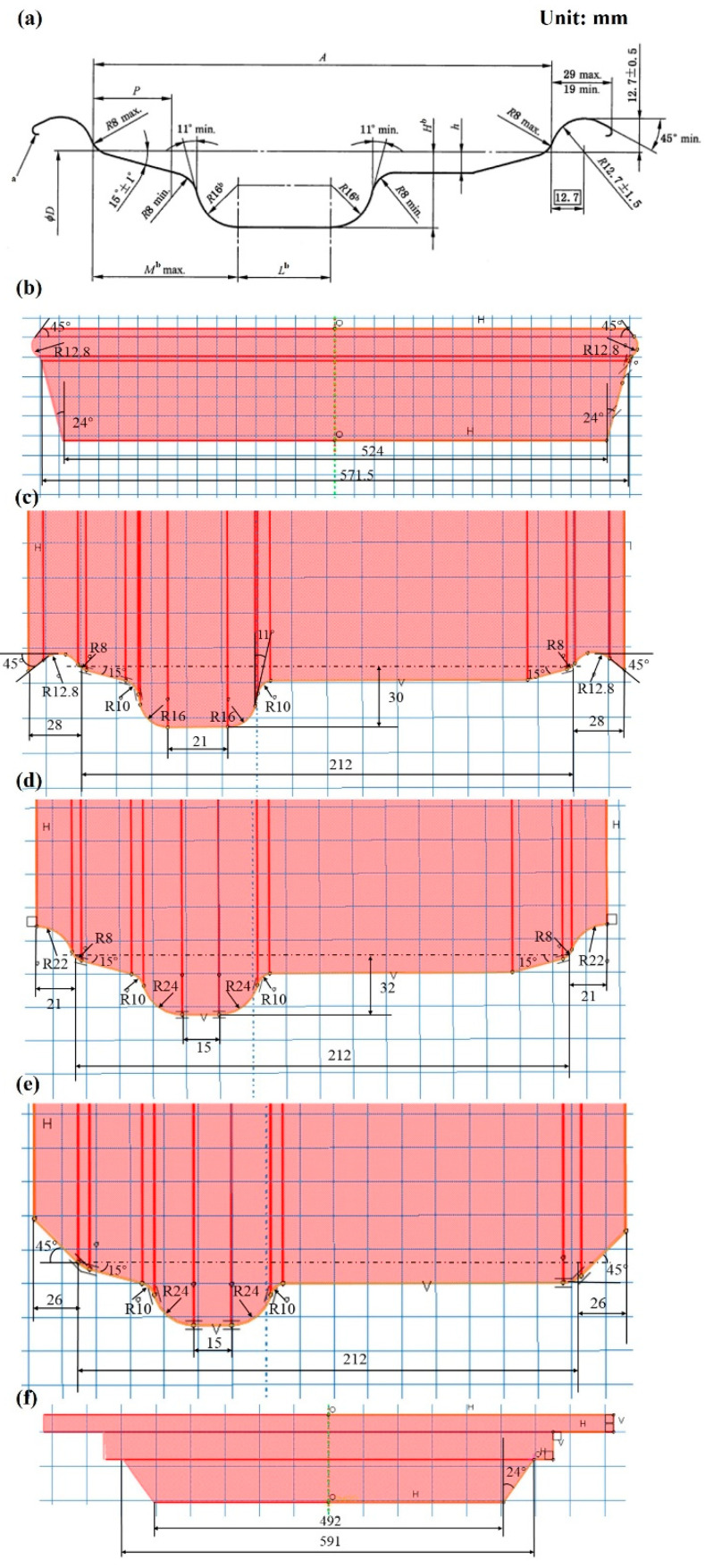
(**a**) 15° deep-groove rim national standard size; mold size: (**b**) expansion die size; (**c**) third-roll die size; (**d**) second-roll die size; (**e**) first-roll die size; (**f**) flaring die size.

The GB/T 31961-2015 [24] profile of a 15° deep-groove rim of 22.5 × 7.50 truck and bus rims, is shown in Figure 2a. It is welded into a ring by plate winding, flaring at both ends, and then formed by three passes of roll bending. The last step is finishing.

In the simulation process, the mold of the rim-forming process can be designed according to the target aim of segmented deformation and the principle of a constant centerline position before and after deformation. The equal gap theory is used to design the size of each pass of the rolling mold according to the final size diagram, as shown in Figure 2b–f.

### 2.2. Finite Element Models of the Roll Forming of the Wheel Rim

The commercial finite element software ABAQUS/Explicit 2020 was used as the simulation tool. ABAQUS/Explicit is a finite element software designed for simulating and analyzing complex nonlinear problems, particularly suitable for fast dynamic events and large deformations. Its primary applications include fast dynamic analysis, large deformation problems, complex contact phenomena, and nonlinear material behavior simulation. It effectively handles high-speed dynamic events such as impacts, collisions, and explosions, providing accurate results for metal forming, material fracture, and collapse, while addressing issues like contact separation, sliding, and friction [25]. It is widely used in the automotive and aerospace industries and in civil engineering [26], manufacturing, and biomedical engineering [27]. However, ABAQUS/Explicit requires significant computational resources during explicit simulations, especially with high-resolution models and long-duration simulations. The choice of time step directly impacts the stability and accuracy of the simulation results. To ensure accuracy, it is common practice to validate and calibrate simulation models using experimental data, and to conduct error analysis to understand potential sources of numerical errors. Through the optimization of parameters and model settings, ABAQUS/Explicit can provide simulation outputs highly consistent with experimental results, enabling in-depth exploration of the nature and principles behind complex dynamic phenomena.

**Figure 3:**
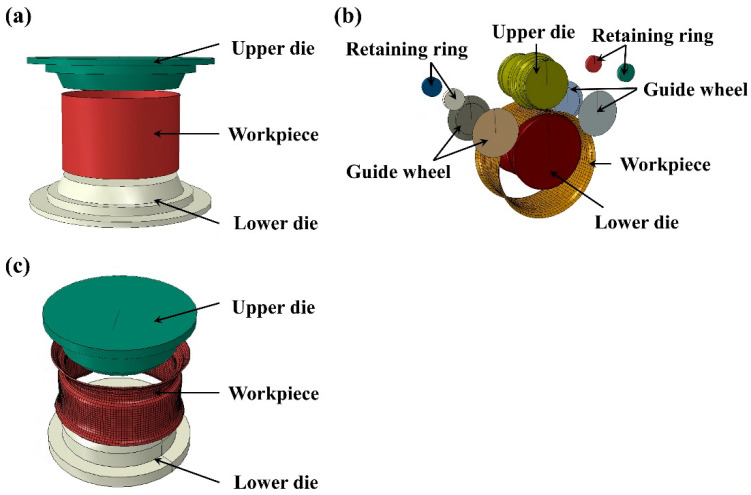
Finite element model of each process of rim production: (**a**) flaring; (**b**) rolling; (**c**) expansion.

Figure 3 shows the model for roll forming. The forming system includes an upper die, lower die, and guide wheel. The upper die and the lower die have convex and concave profiles, respectively. Initially, the flaring ring blank is placed on the lower die. Once the model is started, the upper die and the lower die begin to rotate. The lower die moves upward to contact the upper die, and the annular blank rotates at the same speed as the lower die during the clamping process. The guide wheel and retaining ring are used to stabilize the blank and prevent it from shaking.

**Figure 4:**
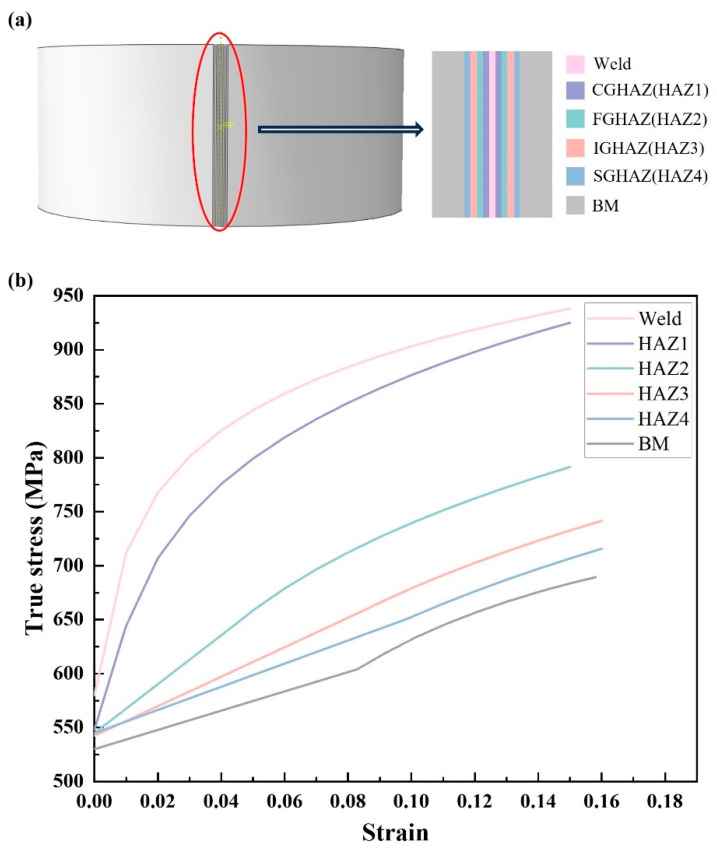
(**a**) Heat-affected zone area division diagram; (**b**) true stress–true strain curves of BM, WZ, and HAZ.

To explore the stress–strain law of the roll forming process of the welded rim, the weld and the heat-affected zone are separately divided when the model is established. Based on the peak temperature of each region, the HAZ can be divided into four main regions: the coarse-grained heat-affected zone HAZ1 (CGHAZ), the fine-grained heat-affected zone HAZ2 (FGHAZ), the inter-critical heat-affected zone HAZ3 (ICHAZ), and the subcritical heat-affected zone HAZ4 (SCHAZ). According to their microstructure and properties, different material parameters are assigned to each region, and a width of 3 mm is set, respectively [28], as shown in Figure 4. The true stress–true strain parameters of each region are introduced after conversion, and the changes in stress–strain and thickness reduction near the weld are observed.

In the process of rim roll forming, three-dimensional finite element modeling is used. Since die deformation during the rolling process can be neglected, the die is modeled as an analytical rigid body. To consider the change in the thickness of the workpiece during the rolling process, the radial force and the axial force are analyzed. In this study, the SC8R solid-shell element was used for meshing, and a total of 4528 elements and 9362 nodes were generated. To ensure the reliability and accuracy of the computational results, we conducted a detailed mesh convergence analysis. We progressively refined the mesh, doubling the number of elements each time, and selected the stress, strain, and deformation at critical locations as the convergence criteria. Additionally, we analyzed the reaction forces and displacements at key points during the rolling process. By comparing the results at different mesh densities, we found that when the number of elements increased to 4528, the variation rate of the stress and deformation at critical locations stabilized, with a change rate of less than 1%, meeting ABAQUS’s convergence criteria. We finally selected a mesh with 4528 elements and 9362 nodes, achieving a good balance between computational efficiency and accuracy. Further mesh refinement showed that the variation rate of the results at critical locations remained below 1%, indicating that the mesh had converged.

In this study, the metal roll forming simulation calculation is carried out using the finite element method. Considering various boundary conditions, such as mold shape, friction, rotation speed, etc., a three-dimensional model is established for nonlinear calculation, and the stress–strain, thickness, material flow, and other related information in the forming process are obtained. The elastic-plastic finite element method takes into account the elastic deformation of the material. It can analyze the dynamic motion of the plastic deformation zone, the stress and strain changes during processing and forming, and can better simulate the actual production and processing process.

Several basic rules of the elastic-plastic finite element method are as follows:

Yield criterion:

When the equivalent stress exceeds the yield stress of the material, the material enters the plastic deformation stage, which satisfies the condition of the yield criterion:

(1) $$F^{0}=F^{0}\left(\sigma_{\mathrm{ij}},k_{0}\right)=0$$

In the formula, $\sigma_{\mathrm{ij}}$ is the stress tensor component, k_0_ is the material characteristic parameter, and F^0^ is the initial yield surface.

When the metal material is deformed, the commonly used yield conditions are:

(a) Mises condition:

(2) $$F^{0}\left(\sigma_{\mathrm{ij}},k_{0}\right)=F\left(\sigma_{\mathrm{ij}}\right)-k_{0}=0$$

Among which,

(3) $$f\left(\sigma_{\mathrm{ij}}\right)-k_{0}=\frac{1}{2}s_{\mathrm{ij}}s_{\mathrm{ij}}\;;\;k_{0}=\frac{1}{3}\sigma_{s0}^{2}$$

(4) $$s_{\mathrm{ij}}=\sigma_{\mathrm{ij}}-\sigma_{m}\delta_{\mathrm{ij}}\;;\;\sigma_{m}=\frac{1}{3}\left(\sigma_{11}{+\sigma }_{22}{+\sigma }_{33}\right)$$

In the formula, f is the plastic stress–strain function, $s_{\mathrm{ij}}$ is the stress component, σ_s0_ is the initial yield stress of the material, and σ_m_ is the normal stress.

The relationship between $s_{\mathrm{ij}}$ and equivalent stress:

(5) $$\frac{1}{2}s_{\mathrm{ij}}s_{\mathrm{ij}}=\frac{{\bar{\sigma }}^{2}}{3}$$

(b) Tresca condition:

In the three-dimensional principal stress space, the yield surface is a regular hexagonal prism with $\sigma_{1}{=\sigma }_{2}={\;\sigma }_{3}$ as the axis and an inscribed Mises cylindrical surface. Its yield trajectory on the π plane is a regular hexagon with an inscribed Mises yield trajectory. The yield function expression is:

(6) $$F^{0}\left(\sigma_{\mathrm{ij}},k_{0}\right)=\frac{1}{6}\left[{\left(\sigma_{1}-\sigma_{2}\right)}^{2}+{\left(\sigma_{2}-\sigma_{3}\right)}^{2}+{\left(\sigma_{3}-\sigma_{1}\right)}^{2}\right]-\frac{1}{3}\sigma_{s0}^{2}$$

Flowing rule:

The relationship between plastic strain increment and the stress state is expressed by Prandt-Reuss:

(7) $$d\varepsilon_{\mathrm{ij}}^{p}=d\lambda \frac{\partial f}{\partial \sigma_{\mathrm{ij}}}$$

In the formula, $d\varepsilon_{\mathrm{ij}}^{p}$ is the plastic strain increment component, f is the plastic stress–strain function, and λ is the proportional constant. this Equation indicates that $d\varepsilon_{\mathrm{ij}}^{p}$ is perpendicular to the surface defined by f = 0.

Hardening criterion:

The hardening criterion of the material indicates that the initial yield criterion changes with the increase in plastic deformation. The hardening criterion specifies the material loading function under plastic deformation:

(8) $$F\left(\sigma_{ij},k,a_{ij}\right)=0$$

In the formula, $\sigma_{\mathrm{ij}}$ is the stress tensor component, k is the plastic work, and $a_{\mathrm{ij}}$ is the moving tensor of the loading surface.

In actual rim processing, to prevent the rim from bouncing or offsetting when it contacts the upper and lower rolling molds, guide wheels are set on both sides of the rim. The guide wheel is mainly used to transfer the pressure provided by the cylinder to the rim. Due to the complex contact between the guide wheel and the edge of the rim, the model of the guide wheel is simplified in the process of numerical simulation, and the force provided by the cylinder is replaced by establishing a spring and adjusting the elastic coefficient and the damping coefficient. According to the calculation formula of elastic coefficient k_max_, the elastic coefficient is set to 1000 N/mm, and the damping coefficient is set to 0.3.

In the flaring process, the upper and lower dies are in contact with the inner surface of the workpiece, respectively. In the three-pass rolling process, the upper die is in contact with the outer surface of the workpiece, the lower die is in contact with the inner surface of the workpiece, and the side guide wheel is in contact with the side of the workpiece. In the numerical simulation, the contact is surface-to-surface contact, and the Coulomb friction model of the penalty function is used. In friction contact, the tangential direction is the friction behavior, the friction coefficient is defined as 0.3, and the normal direction adopts the default ‘hard’ contact. In the simulation process, the rotation speed of the upper rolling die is 180 r/min, the rotation speed of the lower rolling die is 216 r/min, and the feed rate of the lower rolling die is 15 mm/s.

## 3. Results and Discussion

### 3.1. Microstructure and Properties

**Figure 5:**
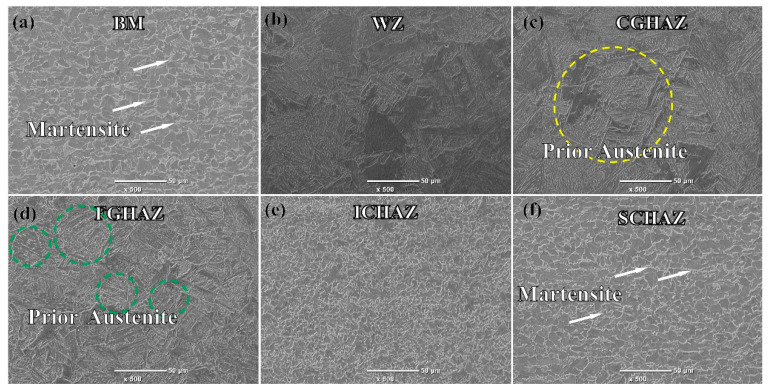
Microstructure of the main heat-affected zones. (**a**) BM; (**b**) WZ; (**c**) CGHAZ; (**d**) FGHAZ; (**e**) ICHAZ; (**f**) SCHAZ.

Figure 5 shows the microstructure of different heat-affected zones at the weld. The microstructure of the DP590 steel matrix metal is composed of 12.81% martensite (M) and 87.19% polygonal ferrite (PF). The WZ has coarse grain and Widmanstatten structure [29]. Using the CALPHAD software “Thermal-Calc” (Thermo-Calc Software, Stockholm, Sweden), the equilibrium phase diagram of DP590 was calculated. The results show that the Ac_1_ and Ac_3_ temperatures are 665 °C and 844 °C, respectively. During the flash butt welding process, the heat-affected zone is divided according to Ac_1_ and Ac_3_: the peak temperatures of the CGHAZ and FGHAZ regions are higher than Ac_3_, leading to complete recrystallization. The grain size in CGHAZ is generally greater than 100 μm, while in FGHAZ it is mostly less than 50 μm. The peak temperature in the ICHAZ is between Ac_1_ and Ac_3_, resulting in partial recrystallization. The peak temperature in the SCHAZ is lower than Ac_1_ but higher than 200 °C, so no austenite transformation occurs, but it still affects performance.

In Figure 5c,d, the CGHAZ and FGHAZ show a lath martensite structure and austenite grain boundaries, with the CGHAZ having larger original austenite grains than the FGHAZ. The ICHAZ consists mainly of fine ferrite and some retained tempered sorbite. The SCHAZ’s microstructure is similar to that of the base metal, but with larger ferrite grains and a lower martensite content.

The average microhardness values for different regions of the weld are: CGHAZ (232.7 HV_0.5_) > FGHAZ (225.8 HV_0.5_) > ICHAZ (187.7 HV_0.5_) > BM (184.2 HV_0.5_) > SCHAZ (167.2 HV_0.5_) [29]. The microhardness of the ICHAZ and SCHAZ is significantly lower than that of the coarse-grained and fine-grained regions. In terms of softening behavior, the hardness of the SCHAZ decreased by 9.2% compared to the BM. The welded heat-affected zone of DP590 steel is defined as an abnormal softening zone that must meet usage requirements. Figure 5 shows that the CGHAZ and FGHAZ have a lath martensite structure, providing high hardness. In contrast, the hardness of the ICHAZ is significantly reduced, mainly due to fine ferrite grains and a small amount of tempered martensite. The microstructure of the SCHAZ is very similar to that of the BM, but its martensite exhibits characteristic tempering, which is the cause of softening in the SCHAZ.

### 3.2. Stress and Strain in the Forming Process

**Figure 6:**
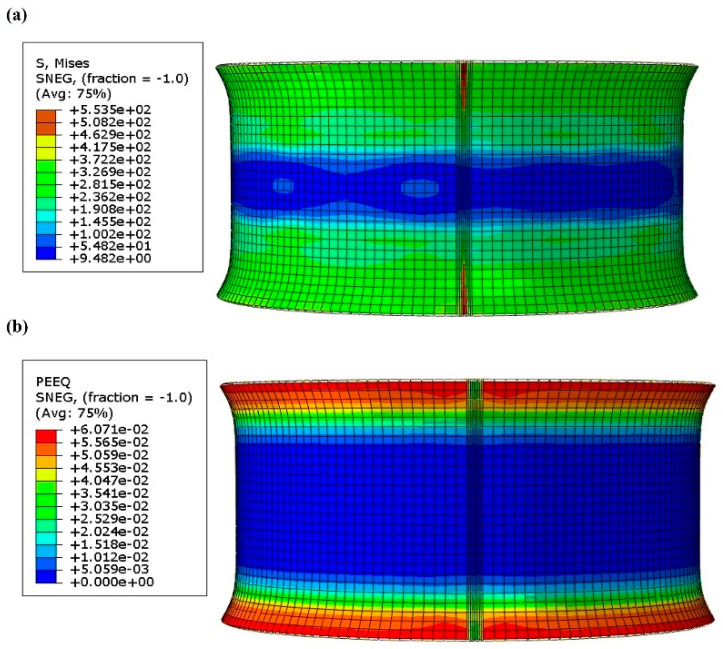
(**a**) Stress distribution mapping of flaring process; (**b**) strain distribution mapping of flaring process.

The distribution of Von Mises stress (S, Mises) and equivalent plastic strain (PEEQ) of the flaring blank is shown in Figure 6. The results indicate that the primary equivalent stress in the workpiece is 372.2 MPa, while the maximum equivalent stress at the weld reaches 553.5 MPa. The majority of deformation during the flaring process occurs at the rim area. After flaring, the rims on both sides of the workpiece take on a horn-like shape, with strain distributed symmetrically. The peak strain observed is 6.07%.

**Figure 7:**
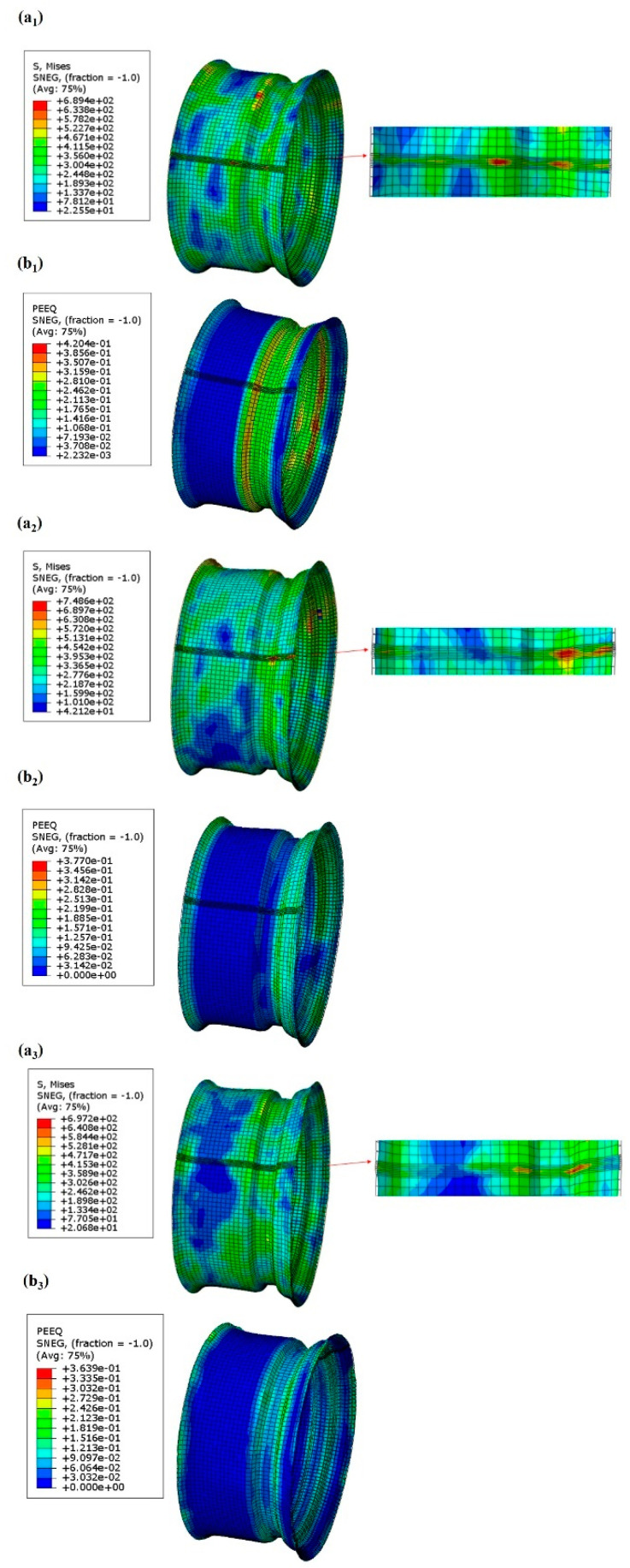
The stress and strain distribution maps of the rolling process: (**a**) stress distribution map, (**b**) strain distribution map; (**a_1_**,**b_1_**) the first pass of rolling, (**a_2_**,**b_2_**) the second pass of rolling, (**a_3_**,**b_3_**) the third pass of rolling.

The distribution of the equivalent stress and plastic strain of the flaring ring formed by the first roll bending is shown in Figure 7(a_1_,b_1_). The weld area experiences stress concentration due to the layered martensitic structure, with the maximum stress reaching 689.4 MPa, primarily localized on both sides of the groove. The coarse grain structure in the CGHAZ and FGHAZ also leads to higher stress during the forming process [30]. The key forming sections in the rolling process are the middle groove and the flanges on either side. The groove is pre-formed, and the flanges are shaped further based on the horn-like form created during flaring. The deformation at the rounded corners on both sides of the groove is the most significant, with a maximum strain of 4.2%, followed by other parts of the groove. The rolling process involves the most extensive deformation in the three-pass process. The plastic strain at the left corner of the groove is greater than at the right corner because, during rolling, material primarily flows from the left side of the groove to the groove’s base, leading to a tendency toward thinning.

The stress and strain distribution of the workpiece during the second rolling process is shown in Figure 7(a_2_,b_2_). Compared with the first pass, the primary goal of the second rolling process is to refine the shape: the groove bottom remains unchanged while the rim is further shaped. In the second pass, the workpiece is mainly subjected to an equivalent stress of about 454.2 MPa. Stress concentration occurs at the fillet weld near the flange side of the groove, which is related to the softening in the ICHAZ and the SCHAZ. These areas are structurally weaker and more prone to thickness reduction. Additionally, the simulation results show significant deformation at the rim near the groove, indicating high radial and axial stress. The maximum stress is 748.6 MPa, correlating with the presence of larger ferrite grains and layered martensite in the microstructure [31]. Regarding the PEEQ, the maximum strain, about 3.77% is found at the groove. The middle groove continues to exhibit substantial strain, with the strain at the left corner of the lower groove exceeding that of the right corner.

After two passes of roll forming, the rim contour is almost formed, except for the corner of the groove. The purpose of the third pass of roll forming is to leave each fillet of the groove and the rim completely formed, and the feed rate is increased compared with the first two processes. Figure 7(a_3_,b_3_) shows the equivalent stress and plastic strain on the forming contour after the third pass of roll forming. In the three-roll process, the rim is first deformed, and then the fillets of each part of the groove is also deformed to varying degrees. The stress concentration occurs at the fillet welds on both sides of the groove, and the maximum stress here is 697.2 MPa. This may be because the material flow mainly occurs in these areas during the three rolling processes, which is consistent with the microstructure in the CGHAZ and FGHAZ, indicating that the layered martensite with a high hardness may be subjected to great stress during the forming process. The maximum PEEQ appears at the rim edge with the thickest contour wall, and the strain is 3.64%.

**Figure 8:**
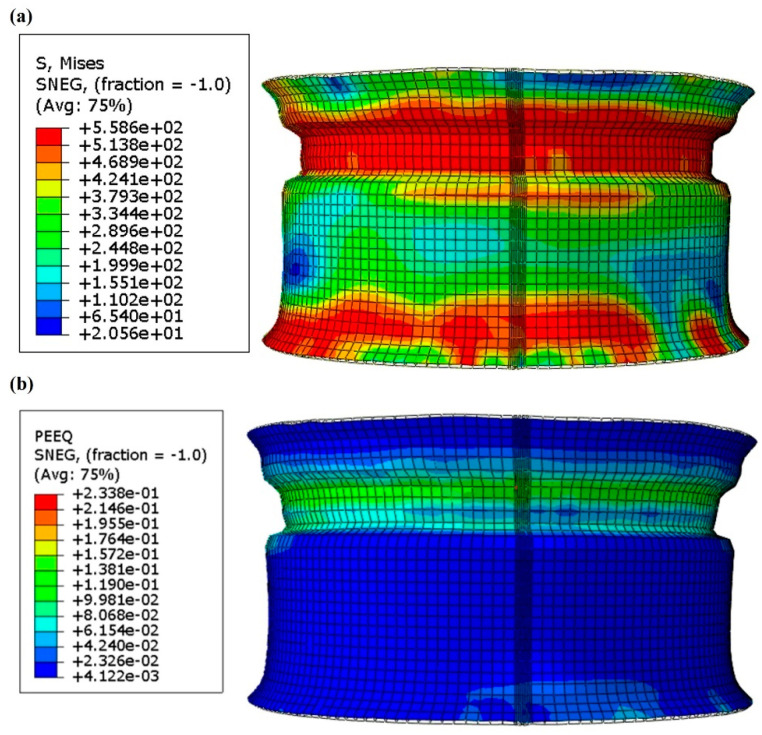
(**a**) Stress distribution mapping of expansion process; (**b**) strain distribution mapping of expansion process.

As shown in Figure 8, the stress and strain distribution of the workpiece after expansion is also called bulging. The expansion process is designed to expand the diameter of the workpiece to the design requirements through the expansion die so that the workpiece flattens. The stress value in the larger stress area is about 558.6 MPa. The strain at the groove is the largest, about 2.33%. In the process of expansion, the diameter of the groove is the smallest; first, contacting the mold and driving the whole to expand. After measurement, the diameter before expansion is 565.8 mm, the diameter after expansion is 571.5 mm, and the expansion rate is about 1%.

### 3.3. Thickness Analysis in Rim-Forming Process

Along the path from the left rim to the right rim, the thickness variation curve of the three-pass roll forming process of the base metal, weld, and heat-affected zone of the rim is output, and the thinning of each region during the rim-forming process is explored.

**Figure 9:**
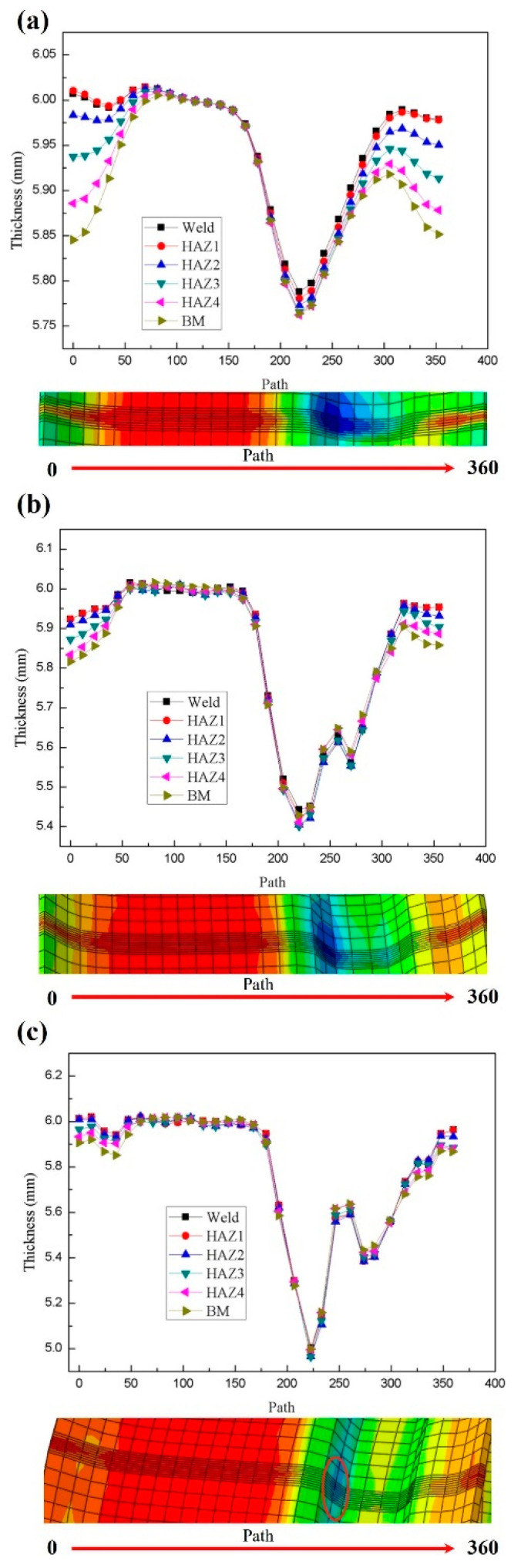
The thickness change curve of different parts of the rim: (**a**) the first rolling; (**b**) the second rolling; (**c**) the third rolling.

The thickness variation curve of different regions of the rim after the first roll is shown in Figure 9a. The main forming parts include the groove and the flanges on both sides. On the left flange, the base material experiences the greatest thinning, with a reduction rate of 3.06%. Secondly, the heat-affected zone HAZ4 has a thinning rate of 2.77%, likely due to the hardness reduction caused by carbon diffusion, martensite decomposition, and tempering of the material. This softening effect may lead to a decrease in hardness in this area, ultimately resulting in thickness reduction. The thickness changes in the weld and HAZ1 are similar, indicating signs of material hardening in these areas. This hardening is primarily due to stress concentration during the first rolling process, which induces work hardening in the material [32]. Since the martensitic structure in the CGHAZ and FGHAZ can provide higher hardness, the rate of thickness reduction in these regions is relatively low. However, the groove exhibits the highest thinning rate, reaching 3.83%, likely due to stress concentration in this area during the first rolling process, resulting in material stretching and deformation.

Figure 9b shows the thickness variation curve of different regions of the rim after the two-roll process. After the second rolling, both the flanges on either side of the rim and the groove experience thinning. On the right side of the groove, near the flange, the thinning is particularly noticeable. This is primarily because, during the second rolling process, the flange and the groove bottom deform almost simultaneously, causing further forming of the flanges on both sides. The right side of the groove is subjected to greater tensile stress, with a thinning rate of 7.33%. This high thinning rate phenomenon may be related to the softening of the structure in the FGHAZ and ICHAZ, making these areas more prone to thickness reduction and stress concentration during the forming process. The thinning pattern in the various regions of the flanges on both sides remains consistent with that of the first rolling. The base material on the left flange experiences the greatest thinning, with a reduction rate of 3.05%, followed by HAZ4 with a reduction rate of 2.77%. However, the differences in thinning across the various regions of the flanges are less pronounced than in the first rolling. The differences in thinning rates in the various regions of the groove are not significant, averaging around 9.67%.

According to Figure 9c, the change in rim thickness after three rolls is similar to that after two rolls, but the deformation is the smallest. Due to the superposition of thickness from the three rolling processes, the thinning at the groove is the greatest, with a reduction rate reaching 17%. This may be related to the martensitic structure in the CGHAZ and FGHAZ, as well as the smaller grain size in the FGHAZ. These microstructural characteristics may lead to greater stress in this region during the forming process, resulting in thickness reduction. On the left side of the groove near the rim, significant deformation from the second and third rolling processes results in considerable tension, with a thinning rate of 9.67%. After the third rolling process, ear widths form on both sides of the flanges, and the difference in thinning rate among the various regions is minimal. This is mainly because the shape of the flanges ensures a relatively uniform thickness distribution.

### 3.4. The Influence of Process Parameters on the Rim-Forming Process

#### 3.4.1. The Influence of Friction Coefficient on the Forming Process

In roll forming, the rim rotates with the upper and lower molds by friction. However, due to the influence of lubrication conditions and mold surface quality, it is difficult to determine the relationship between the friction and the sensitive parts. According to the empirical values in the literature, the effects of friction coefficients of 0.1, 0.2, 0.3, and 0.4 on the forming quality of the rim were investigated in the simulation.

**Figure 10:**
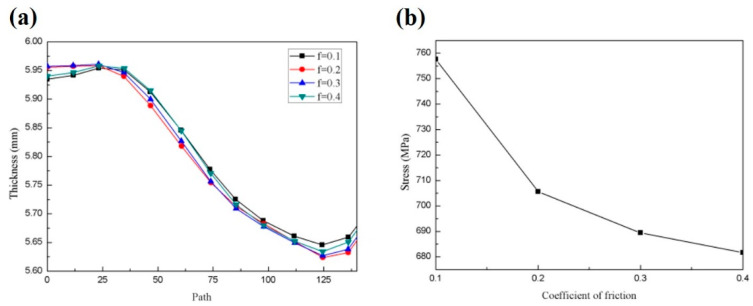
(**a**) Effect of different friction coefficients on rim reduction; (**b**) effect of different friction coefficients on equivalent stress.

Since the weld seam at the groove of the rim is the most obvious area of thinning, the thickness change in the weld seam after one roll forming pass with different friction coefficients is mainly studied. Figure 10a shows the influence of different friction coefficients on the thickness of one side rim, and the path from left to right is rim-to-groove. It can be seen that when the friction coefficient is 0.1, the flange thickness is smaller and the groove thickness is larger; when the friction coefficient is 0.2, the thickness of the groove is the smallest and the thinning rate of the groove is the highest. The thinning effect of a friction coefficient of 0.3 is similar to that of 0.2. Because groove forming involves material distribution and flow, too small a thickness will lead to an excessive thinning rate, and balanced thickness distribution is more conducive to forming quality. Figure 10b shows the influence of different friction coefficients on the equivalent stress. The higher the friction coefficient, the smaller the equivalent stress and the lower the forming difficulty. Based on the above results, the forming quality is best when the friction coefficient is 0.4.

#### 3.4.2. The Influence of Feed Rate on the Forming Process

The feed distance refers to the distance during which the lower roll feeds upwards, contacts the inner surface of the workpiece, and continues to drive the workpiece upward until the outer surface of the workpiece contacts the upper roll. If the feed speed is too high, it can easily cause the workpiece to “deviate”, resulting in asymmetrical wheel flanges after forming. Additionally, significant material flow occurs in the groove formation during the first rolling process. Once the groove position is determined, it lays the foundation for subsequent processes, making analysis of the quality of the first roll forming critical. The feed speed affects the force exerted on the workpiece by the mold; excessive force increases the difficulty of the rolling process and destabilizes the model.

**Figure 11:**
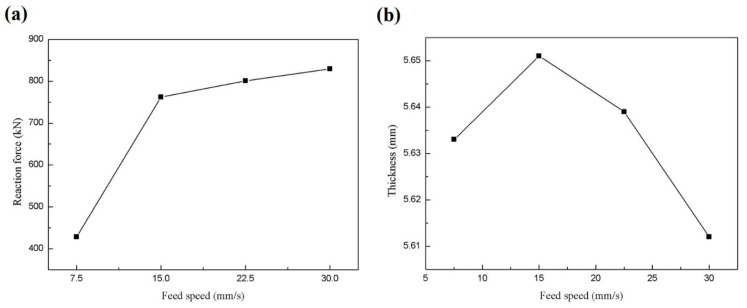
(**a**) Curve of the reaction force at different feed speeds; (**b**) curve of minimum thickness at different feed rates.

The variation curve of the maximum support reaction force of different feed speeds obtained by simulation is shown in Figure 11a. The back force represents the interaction force between the workpiece and the guiding wheel, reflecting the stability of the forming process. As the feed rate increases, the model experiences higher back forces, indicating increased difficulty and instability in the forming process. In practical production, prolonged exposure to significant loads and impacts can prematurely fatigue equipment components, reducing equipment stability and accuracy, and potentially causing the wheel rim forming to deviate. Figure 11b illustrates the variation curve of the minimum thickness of the wheel rim at different feed rates. It shows a trend where the minimum thickness initially increases and then decreases with an increasing feed rate. A larger minimum thickness indicates less thinning of the wheel rim during forming, indicating higher forming quality. When the feed rate increases to 30 mm/s, the thinning rate reaches 6.47%, accompanied by higher back forces, which are unfavorable for stable forming. Considering the impact of feed rate on back force and thickness, a feed rate of 15 mm/s provides better stability for the forming model.

#### 3.4.3. The Influence of Mold Speed on the Forming Process

When the rim is rolled, the down roll of the driving wheel drives the workpiece to rotate, and its speed is larger than the up roll, which can make workpiece forming easier. The mold speed affects the initial speed of the rim starting to rotate. Taking 180 r/min as the up-rolling speed, ratios of 1:1, 1:1.2, 1:1.5, and 1:1.8 were set up to study the influence of down-rolling speeds of 180 r/min, 216 r/min, 270 r/min, and 324 r/min on the rim-forming process.

**Figure 12:**
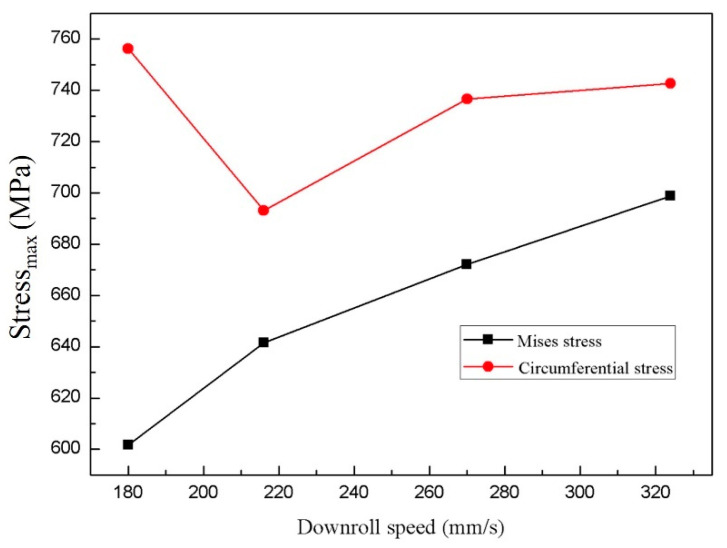
Effect of different rolling speeds on equivalent and circumferential stress.

The speed of rolling down will affect the initial speed of rim rotation and the stability of the model. Figure 12 shows the effect of the down rolling speed on the maximum equivalent stress and circumferential stress. The circumferential stress refers to the stress acting on the rim circumference (the direction around the rim). These stresses are caused by the pressure applied in the forming process, the transfer of force, the deformation of the material, and the effect of the forming die. It can be seen from this Figure that the equivalent stress extremum increases with the increase in the rolling speed. The equivalent stress extremum of the one-roll forming process is mainly concentrated near the fillet of the groove. An excessive stress value can easily lead to stress concentration, making it a dangerous part. From the extreme value of circumferential stress, when the rotational speed of the lower die is 180 r/min, the extreme value of circumferential stress is at its largest, reaching 758 MPa. An excessively low rotational speed increases the difficulty of forming, and excessively high circumferential stress will increase the likelihood of circumferential cracking of the rim. With the increase of rotational speed, the circumferential stress extremum decreases first and then increases. When the rolling speed is 216 r/min, the circumferential stress extremum is at its lowest, which is 692 MPa. When the rotational speed continues to increase, the extreme value of circumferential stress increases again, but the change is small. In order to reduce the difficulty of forming and ensure that the stress on the rim cannot be too great, a ratio of up- and down-rolling speed of 1:1.2 is the most suitable.

## 4. Conclusions

This study uses finite element simulation to analyze the stress–strain distribution, thickness reduction, and microstructural changes during the rim roll-forming process of high-strength dual-phase steel DP590. The simulation results are consistent with the expectations, and the main findings are as follows:

1. Stress and Strain Concentration: Stress and strain are mainly concentrated in the welded region and heat-affected zones (especially the CGHAZ and FGHAZ), leading to local deformation and thickness reduction.

2. Thickness Reduction: Thickness reduction primarily occurs in the groove and flange sections. The reduction rate in the groove section is 3.83% after the first rolling step, 7.33% after the second step, and 17% after the third step, mainly due to material softening and carbon diffusion in the heat-affected zones.

3. Role of Microstructure: Microstructural characteristics significantly influence stress and strain distributions. The layered martensitic structure in the CGHAZ and FGHAZ causes high stress, while softening in the ICHAZ and SCHAZ leads to thickness reduction. Coarse grain and Widmanstatten structures in the welded area also contribute to stress concentration, affecting forming stability.

4. Parameter Selection: In the roll forming process, a higher friction coefficient can improve forming quality, and a friction coefficient of 0.4 is recommended. Additionally, the proper feed-speed and roll-speed ratio can enhance stability and stress control, with the optimized match by the feed speed of 15 mm/s and a 1:1.2 ratio of the upper and lower roll speeds.

This study provides new insights into the key regions and microstructural characteristics of high-strength dual-phase steel DP590 during rim roll forming. By optimizing process parameters and conducting in-depth investigations into microstructures, the manufacturing quality and stability of rims can be enhanced. Future research directions can focus on the following areas:

1. Expansion into application fields: The successful application of dual-phase steel in rim manufacturing opens up potential for its use in other mechanical structures. Particularly in the automotive and aerospace industries and in rail transportation sectors, the high strength and excellent formability of dual-phase steel can significantly improve the strength and durability of structural components.

2. Exploration of new simulation methods and material types: Future studies could introduce multi-physics coupling simulations and advanced numerical optimization methods to optimize parameter selection and process design in rim-forming processes. Similarly, similar analytical techniques could be applied to other advanced high-strength steel materials, such as improved models of dual-phase steel or other complex structural alloy steels.

3. Further improvement of material performance: Research efforts could focus on enhancing mechanical properties and forming quality of dual-phase steel through microstructure control and process parameter optimization. This includes optimizing forming processes to reduce stress concentration and thickness reduction, thereby improving product stability and reliability.

## Figures and Tables

**Table 1:** DP590 alloy elements and mechanical properties.

Alloying Element	Elements	C	Mn	Cr	Si	Al	Ti	Nb	P	N	S	Fe
	wt.%	0.056	1.199	0.268	0.085	0.039	0.02	0.013	0.015	0.004	0.001	Bal.
Performance parameter	Yield strength	318 MPa										
	Ultimate tensile strength	614 MPa										
	Elongation rate	39.3%										
	Young’s modulus	199 GPa										
	Poisson ratio	0.33										

## Data Availability

The original contributions presented in the study are included in the article, further inquiries can be directed to the corresponding authors.
